# Supplementary material for: Structural insights into target detection by the S. marcescens type III CRISPR complex and its deployment in SNP identification
Source: bioRxiv. 2026 Mar 31:2026.03.30.715313. Preprint. [Version 1] doi: 10.64898/2026.03.30.715313 (PMC13060164; doi:10.64898/2026.03.30.715313)
Supplement: Supplement 1 [file media-1.pdf]

Structural insights into target detection by the *S. marcescens* type III CRISPR complex and its deployment in SNP identification

Calvin C. Perdigao[1], Luqman O. Ajisafe[1], Anju T. Sunny[1], Si Wu[1], Terje Dokland[2] and Jack A. Dunkle[1]*

[1] Department of Chemistry and Biochemistry, University of Alabama, Tuscaloosa, AL, USA

[2] Department of Microbiology, University of Alabama at Birmingham, Birmingham, AL, USA

*Corresponding author: Jack A. Dunkle

E-mail: jadunkle@ua.edu

Supporting Information

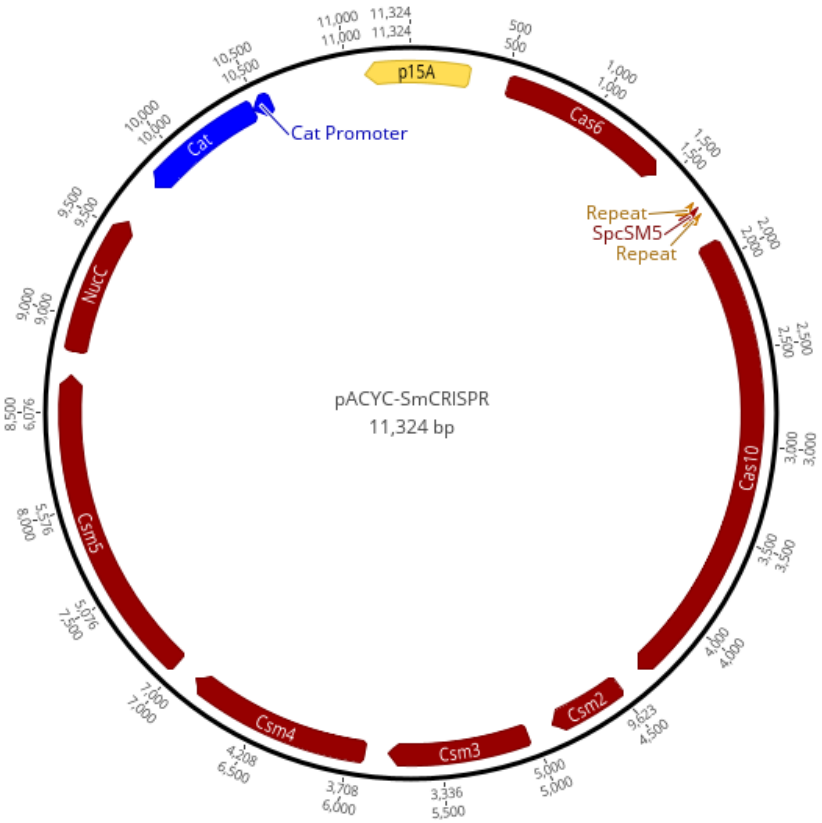

Figure S1. Plasmid map for pACYC-Csm

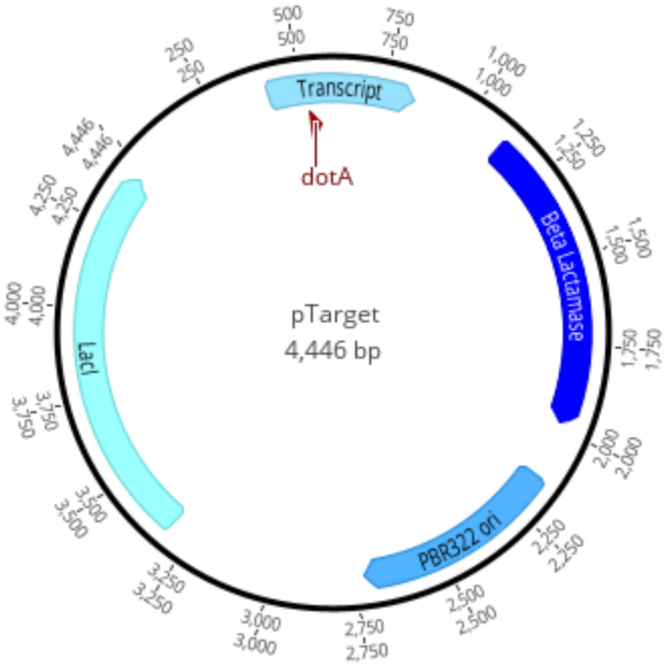

Figure S2. Plasmid map for pTarget

Table S1. Plasmid sequences, gene fragments and oligos used in the study.

Name	Sequence (5’-3’)
Plasmids	
pACYC-Csm	1 atgcacgaac cccccgttca gtccgaccgc tgcgccttat ccggtaacta tcgtcttgag<br>61 tccaacccgg aaagacatgc aaaagcacca ctggcagcag ccactggtaa ttgatttaga<br>121 ggagttagtc ttgaagtcat gcgccggtta aggctaaact gaaaggacaa gttttggtga<br>181 ctgcgctcct ccaagccagt tacctcggtt caaagagttg gtagctcaga gaaccttcga<br>241 aaaaccgccc tgcaaggcgg ttttttcgtt ttcagagcaa gagattacgc gcagaccaaa<br>301 acgatctcaa gaagatcatc ttattaatca gataaaatat ttctagattt cagtgcaatt<br>361 tatctcttca aatgtagcac ctgaagtcag ccccatacga tataagttgt aattctcatg<br>421 tttgacagcc ttcagatccg atagactagc cgctggtaat aatacgactc actataggga<br>481 gagaattcta agaccgaaag tcggaaacaa agaggattta tatgacatgg gtttgccatc<br>541 tggcgcgtta ccgcctctgt tttcaaagca tcgaaccgct ggcgctgccg ctgtttagcg<br>601 gctccatgct gcgcggcgcg ttcggccatg cgctgcggcg catctgttgc atcagccgtc<br>661 agaaacagtg tgacggctgc ccgttgctgg ccggttgcca gtacccgctg ttgtttgagc<br>721 cgcgcttgct gcaaaacacc cccggccagc cccaaccggc tccgccttat gtgctggagc<br>781 cagcggccgt tcagagggag cttgccgcag gggaaatctg gccggtagac gtggtgctgc<br>841 acggcccggc gctaccgcac ctgagcctga ttattctggc ctggatgcag gcagctgttc<br>901 aggggtttgg cagccagcgg gtgccagcac agttgtgcca ggtgcaggta gagcaacccg<br>961 atactgaagc gcgctggctg acgatctggc gtcacgacca gccgtttatc ctgccacacg<br>1021 ccaccacgca aacaccgcca ccgcccccgg cggcagaggc gctgcgcctg cacttcacca<br>1081 ccccgacccg gctgttgcgc caggggcaac tggtacgggt gcgtgaactt caggcacacg<br>1141 acctgctggc cgcgctggag cgacgcctgc acacgctggc cgcctcgctg ggcattgccc<br>1201 cgccgccgtc gctgaccacg caactcgtta cgctgcaacc cggccagttg cgctggatga<br>1261 actggcaacg ctactccagc cgccagcaac aggagatgaa tctcggcgga tttatcggtg<br>1321 acgttacgct ccacggcgag ctgacgccgc tgtggacatg gctgtggctg ggccagtggt<br>1381 tgcatgtggg caaaaacagc agcttcgggc tgggacgcta tcagttagag acggttcccc<br>1441 gctaaaagct ttcctgtgag cagcgaaagc ctagcataac cccttggggc ctctaaacgg<br>1501 gtcttgaggg gttttttgtt atacgcgaga taatcacttg catagctgcg tatggaggaa<br>1561 gcaactcttg agtgttaata tgttgacccc tgtattaggg atgcgggtag tagatgtggg<br>1621 cagagacacc cacactgcca gatcttaata cgactcacta tagggagacc atgggtcctt<br>1681 acggacgctc cctgactgaa gggattaaga ccatttacag aacctgttcc acttaatgaa<br>1741 gttgcgtcct tacggacgct ccctgactga agggattaag acctcgaggc tgtggtctag<br>1801 acattccata catatcgggg gggtaggggt tttttgtgtg cctctagtgg ctggctaaga<br>1861 ataatacgac tcactatagg gagaggatcc ataaaggagg taaataatga actggcttgc<br>1921 cgcctcttgc catgtggctg cctttgcgct gttacataac ctgaaaccgc tggcgcagcg<br>1981 tgccggcatt caggatttcc cgtcaccggc gctggcaaac aaactgtttg cgccgctgcc<br>2041 ggaaagcctc tcggcccgtg cggatctgca cgaaaataca ttaaccctgc tattggattt<br>2101 cgccgccaga ctggcgcgcg gcctgccgga gacgcccgta agccgtggcg aaaccgggct<br>2161 gataccgctc acccacctgt tagcggacga taaccacagc gccccggcgg agcgctgtta<br>2221 cagcccactg gccccgctgg gggtcgattc gctgatgccg gtgacggcag cgttgcccac<br>2281 cggcgaacgg caggcggcgt accggcaggt gtatcaggca ttgatagagg ggctggaggc<br>2341 gatccccgcc gcccaccgcc tgcaaccgtc gctgtggctt gaccacctcg acagcctgtg<br>2401 gatgaccacc tgccacgcgc tgccagacgg cgacggggcc agcggtatct cgctgtacga<br>2461 tcagggcaaa accacggcgg cgctcgccgt cgcactgtgg cagcaccacg cccgtcaggc<br>2521 cacaccggag aaggaaattt cactcacgcc aggcgacgat gcgcgcctgc tgctgattca<br>2581 ggccgatgtg ttcggcattc aagagctgat tttcgcccag ggcaatcaga cgcaaaaaat<br>2641 ggccgccaaa ctgctgcgcg ggcgttcgtt tcaggtatcc ctgctggcgg aaaccgctgc<br>2701 gctgcgggtg ctggaaacct ttgacctgcc gccggtttgc cagttgatta acgccgccgg<br>2761 caaaagcctg attgtcgcgc cgaatctgcc ggatgccgcc gaacggctgg ccgggttgcg<br>2821 tcagcgtctt gatgagtggt ttttaaccca cacctacgcg caaaccggca tcgggctgtg<br>2881 cagcacggtg gccggcagcg ctgagttcct cggcgagcag gcttacggca ggctgcaaaa<br>2941 ccggctggcg caggcgatgg agcaacagaa ataccagcgt tttccctgt gtgccggtga<br>3001 tgcgcccccg ccggtgtttg agggttatct ggaccaaata gcgcagggcg caggcggtga<br>3061 accctgcccg atgaatggcc tgcacccggt ggaaaccacc ctcaacgggc ttggctgtag<br>3121 ccgactggcg gcagaccaga taaccctcgg ccactggata aaccacggcc actggataag<br>3181 ccacggcgag cgttacgccc ggctgttgat cctgcgtgac agcaccgcca ccttctctga<br>3241 tgaccgctgc ctgcacctga cgctgtttgg ctatcaggtg gtggccgtca gcctgaaaga<br>3301 ggacagcggc gagttcggcg aactggcgtg caacggcagc ctgcgccgct gctgggatgt<br>3361 cagcctgccg ccggaaggca cctccctgtt tcaggttat gcccgccgtt ttatcaacgg<br>3421 ctggatgccg ctggcgcagg gggaatatca gccggagtgc taccccggca ttgaagaagc<br>3481 gctcgctaac ggcgacatca aaacctttga tcacctgagc tgtgaagatt tgtatcagga<br>3541 tgccgaccag tcgttacgcg gcacctgcgc gctgggcgtg ctgaaagggg atatcgacaa<br>3601 cctcggccac ctgtttcgca gcggcctgcc gcaaccgggc tttgccaaaa ccatcggcct

		3661 gtcgcgccag atacacctgt ttttcactct gtggctgccg catctgtgcc gcaaagaccc<br>3721 gcgttttgcc aacacctaca ccgtgttcgc cggcggcgac gatttctttt tgattggccc<br>3781 gtggcgcagc cagcaaacgc tggcgctaac aatggcgcag gacttcgcgc gctacagcgg<br>3841 ccacaatccg gcgctgcatt tttcactcgg gctggtgcag gccaaacccg gctacccggt<br>3901 gcgggcgctg gcggctcagg ccgaagccgc tctgaagcag gccaagcaac accccggcaa<br>3961 gaacgccatc tgcctgtata acgaggtgat gggctggccg gaatatgacg cgttactggc<br>4021 gtgcagtgag gagctggcgc gctggcgcga gcatgacggc tacccgctct cctccggcct<br>4081 gctttaccgg ttgctggcgc tcagtgaaca gtcggcggag gagtcagaaa aaccgcaggc<br>4141 ggcactgtgg cgcagccggc tggcctattt tttacgtcgc aatctggtgg ataacgtgaa<br>4201 agtcccgacg aaagaaaacc cggcagcgtt ccgccatcag cttcatttgg aattgtttga<br>4261 gaaacttgag cagcacctga aacggcaccg ccagcgttac cgggtggcgc ttcaacgcca<br>4321 tctctaccac taccgcacgg tgccgcgcgg cgagtaacat atggctgcgt ggtcaaatgt<br>4381 gcgtacccta accccttccc cggtcaatcg gggcggatgg ggtttttttgt gcgtacttca<br>4441 ttatgtatat taatacgact cactataggg agaagatcta taaaggaggt aaataatgca<br>4501 tcaccatcac catcacacga cgctcgacta ttttaaacgc caactgcaca agccggaggc<br>4561 gacatttttt gatgacgacg cccaaaaatt tgccgaacag ttggctcaat acggtaagaa<br>4621 gggcaaaccc actcagttgc gccgctttta cgaccagtta cagcaactgg agcagcgcat<br>4681 caatggcgat gaggaaaaac tggctctcta cctacccgag atccgcatga tcagcgccca<br>4741 tctggcgtat gccaaagggc gcgagcttat ctctgacgag ttttgccaaa ccatgcaaaa<br>4801 cctgattcgc agcatcaaca cctgccagca cctgaaaaat ggccggctgt tttttgaagc<br>4861 caccctcggt tttttacgcg ccatacgccg cgactgaacg cgtgctgcgt ggtcaaatgt<br>4921 gcgtagacca accccttgcg gcctcaatcg ggggggatgg ggttttttgt caggcaagtc<br>4981 tcagctggtt taatacgact cactataggg agagaattca taaaggaggt aaataatgca<br>5041 actgaacaat atccagacgt tacgcgccac tctggtgtgt gaaaccgggt tacacattgg<br>5101 cggcggcgac accgcgttgc agattggcgg catcgacagc gccgtggtgc gccacccgtt<br>5161 gacccagcaa ccttacattc ccggctccag cctgaaaggc aaactgcgca gcctgctcga<br>5221 atggcgcgct ggtgtggtcg gcgataccga gggcaaggtg ctcagtcatc aggtttacca<br>5281 gcaactgatc gacgataaaa aacaggcaca ggcactacag gcactgaaaa tcctgcaact<br>5341 gtttggcgtc agcggcggcg acaagctttc cgccgaacaa gcccaacaga tcggcccgac<br>5401 tcgcctgtcc ttctgggatt gcgaatttga cgaacactgg ctggcgcagc aaggcgggcg<br>5461 cgtccagacc gaagagaaag cggaaaactg tattgaccgc atcagtggcg tggcgctgca<br>5521 cccgcgcttt atcgagcgtg tgcccgccgg cagccgcttt gactttcgcc tcaccgtgcg<br>5581 ccagcttgat ggcgacagcc ccgacctgct cgacaccctg ttggcgggcc tgaaaatgct<br>5641 ggagctggac gggctaggcg gcagcatttc ccgtgggtat ggcaaagtgc gctttgaagc<br>5701 gctgaccctc gacgggaaag atctccagcc gcgctttgag cagttacagc cgtttaaaca<br>5761 caccacgcag ggagccggat gaaagcttac ctggagatca aggagattac tctaacccca<br>5821 tcggccgtct taggggtttt ttgtcctgtg ttagctggag ggtataatac gactcactat<br>5881 agggagaccc gggataaagg aggtaaataa tgccctcccc cgttgccccc gccgccgcat<br>5941 ggcaatggct acgcctgcgg ctgctccccg acagcgcctt tgccaccgcg ctgcgcggtg<br>6001 acacgctgtt tggccagtta tgctggtatt tgcgcgaatc cctcggtgag gcgggcctga<br>6061 atgcgctgct ggcaggctac cacgaccagc gaccgttcgc ggtgatcagc gacccgatgc<br>6121 tggccgacca cctgccgcgc ccgcacctgc ccgaacaccg gctgggcttc gccgccgccg<br>6181 atgcccgcgc ccgcaaacaa cgtaaacagc aatgctggct gccgcttgcc ttcgctcacc<br>6241 agccgctggc cgagtggggc gcacacttaa ccgccgcccc tgagcaccac cagcaccgca<br>6301 gcgacgtgca gatgcacaac agcatcaacc gccagacact taccaccggt ggcgatgacg<br>6361 cgttcgcccc gtttggcagc gagcagcact ggtttgatgt ggacaccgcc tgggatctct<br>6721 tggcagacag cgccgccgtg ctggcccccg ttaccccgga taaccgcccg tttgtcggcc<br>6781 aggggattagg cggcaatggc cgtctttcca ccgccctgcc acaaacggtg catcagggct<br>6841 atgcgccggt gatcccggtg cgctttcacc ataaggcaca gccacaatga tcatgatttc<br>6901 ttgtcgaact ggacagtagc agaaccgcta acgggggcga aggggttttt tgtgacatac<br>6961 gagctgattg aactaatacg actcactata gggagaggta ccataaagga ggtaaataat<br>7021 gacccgcgac cagacgccac gtcgccacac cgcctgtgac agcgacaaca tgcaatactt<br>7081 taccctcacc tgcctgtcgc cggtgcatgt cgccaccggc gacagcctga accccggtga<br>7141 atacctgata gacgaaaatg cactgtatga actggggcaa ggtggtctga gcccggcgct<br>7201 cacggcgaca cagcgcagcg agttgctcac tattctggag agcaatgacc ctgctctgcc<br>7261 gctgaccgtg cagcgttttc tggcacggga agcgggcaag ctgaaatacg ccgcccgccg<br>7321 gatgtgcccg ctgttacccg gcatcagccg ttattaccag tcacggctcg ggcaggtgat<br>7381 gcagaacgat accaaaaaca aaaagcagat gatcaaccag ctagagctga tgcgccacgt<br>7441 cggggccgca ctgggcgcgc cttatattcc cggatctacg ctcaaagggg ctatccgcac<br>7501 cgcactggtc agcgcgctca atcaaggcca gccactgcaa gccgagcgac gggaaaccga<br>7561 caaactgagc agcaaggtgg cgcaggatgc ggaacgccag ctactgggct ttgatacccg<br>7621 ccgcgacagc ccgcgatacc gcattgagca tgacccccttc cactggctac aggtgggggga<br>7681 tgccgtaagc ccggcagagc acccgcccat gctggattac tggctggtac gccgccagcc<br>7741 gttcaaacgc accgagaagc aggacaacaa ggcggacaat atggaactgt cgccggttga<br>7801 atgccttaaa ccccggcaaa gcccgttgca ctgccagata accgtgaaaa cgccgcccac<br>7861 cgctctcgca ataaaaaacc cgcgcctgaa gcagtggctt ggcaaggtgt cgcaactggc<br>7921 gcaacaggtg aacaggataa ccctgccgca atgccatcac gagctggcat ggctggccga<br>7981 aaagcacatc ggcacggatg atgtgtacgc ccccggccag aactgggtgg cgcagatgca<br>8041 gcaattactg cgccagttag acgacccgct acagcgcggc gaggcactct tgctgcgggt

	8101 gggcaaatac ggcggagcca tcagcaaaac cgtggcaggc tggcggcata tcgcccggct<br>8161 ggggagacaa ggcacgcgca ccacctacca cccggacgtc accacctgca cgctggcgct<br>8221 gccgcaagct gatgcgctga cacaggcgct gcccctttggc tgggtactgt tgcaccagcc<br>8281 tgaccagccg gaggtgaccg agtttgtcgc cagccatcat gactggtgcc agcaacagca<br>8341 acagcggctt gacgcgcatc agcaacagca gcacacccac cgccagcaac gccagcagtt<br>8401 ggctcaggct cgtgaagagg aagcgcagcg gctggcagac aaagcccgcc agagcaaagc<br>8461 gcgccagtcc atcatgtcgc tggcagaaca actggcgagt gagcaaacgt ttcagcataa<br>8521 aaaccccaac ggcccgctgc gcggtcagtt agccacctgt gtgggttgcg ttgccactga<br>8581 aggctcagca gaagagaaag ccgagttgtg cacactgttt gacgacatcc tcaactattg<br>8641 gggcatcaaa cccggcaaag ataaaaagct cacggccttg aggaataagc tgttatgacc<br>8701 taggcgcttc aacggaacgg atcttacata tcggggggt aggggttttt tgtctcggag<br>8761 accaagtagg gcataatacg actcactata gggagactat ggataaagga ggtaaataat<br>8821 gactaatcag gcaaaaaagt tatctagaat taatggtagg gagtttttaa aacagtcctt<br>8881 taatttacaa caacaactat tggcctctca attaaattta tcccgaacga ttacgcatga<br>8941 tggaacgatg ggggaggtta atgaaagtta ttttttgagt attatccgcc agtatttgcc<br>9001 tgaacgttac tcggttgacc ggggagttgt ggtggattca gaaggccaga ccagcgacca<br>9061 gatagatgca gtgattttttg accggcatta cacaccgaca ttattagacc aacaagggca<br>9121 caggtttatt ccggcagagg cggtgtacgc ggtactggag gttaaaccaa ccattaataa<br>9181 aacctacctt gaatatgcag ccgataaagc tgcatctgtc cgaaaattat atcgaaccag<br>9241 tacggtaata aaaaatattt acggtacggc caaaccggtc gaacatttcc cgatcgtagc<br>9301 aggtattgtg gcgattgatg ttgagtggca agacggactc ggaaaggcat ttactgaaaa<br>9361 tttgcaggct gtttccagcg atgaaaaccg aaaactggat tgcggtctgg cggtgtcggg<br>9421 cgcatgtttt gatagttatg atgaggaaat aaaaatcaga agcggtgaaa atgcattaat<br>9481 ctttttttctg ttccgtttgc tcggtaaatt gcaatcatta ggtacggtgc ccgcaattga<br>9541 ctggcgggtg tatatagata gtctggaata actcgagagg ttacagcctg cataatgtag<br>9601 cataacccct tggggcctct aaacgggtct tgaggggttt tttgtgccta tagtttgaag<br>9661 cagaatcgaa tttctgccat tcatccgctt attatcactt attcaggcgt agcaccaggc<br>9721 gtttaagggc accaataact gccttacaaa aaacccctag ccgcccgata agagcgggct<br>9781 aggggttcga gtaaaaaaaa ttacgccccg ccctgccact catcgcagta ctgttgtaat<br>9841 tcattaagca ttctgccgac atggaagcca tcacagacgg catgatgaac ctgaatcgcc<br>9901 agcggcatca gcaccttgtc gccttgcgta taatatttgc ccatcgtgaa aacgggggcg<br>9961 aagaagttgt ccatattggc cacgtttaaa tcaaaactgg tgaaactcac ccagggattg<br>10021 gctgaaacga aaaacatatt ctcaataaac cctttaggga aataggccag gttttcaccg<br>10081 taacacgcca catcttgcga atatatgtgt agaaactgcc ggaaatcgtc gtggtattca<br>10141 ctccagagcg atgaaaacgt ttcagtttgc tcatggaaaa cggtgtaaca agggtgaaca<br>10201 ctatcccata tcaccagctc accgtctttc attgccatac ggaactccgg gtgagcattc<br>10261 atcaggcggg caagaatgtg aataaaggcc ggataaaact tgtgcttatt tttctttacg<br>10321 gtctttaaaa aggccgtaat atccagctga acggtctggt tataggtaca ttgagcaact<br>10381 gactgaaatg cctcaaaatg ttctttacga tgccattggg atatatcaac ggtggtatat<br>10441 ccagtgattt ttttctccat tttagcttcc ttagctcctg aaaatctcga taactcaaaa<br>10501 aatacgcccg gtagtgatct tatttcatta tggtgaaagt tggaacctct tacgtgccga<br>10561 tcaaggtctc attttcgcca aaagttggcc cagggcttcc cggtatcaac agggacacca<br>10621 ggatttattt attctgcgaa gtgatcttcc gtcacaggta tttattcggc gcaaagtgcg<br>10681 tcgggtgatg ctgccaactt actgatttag tgtatgatgg tgtttttgag gtgctccagt<br>10741 ggcttctgtt tctatcagct gtccctcctg ttcagctact gacggggtgg tgcgtaacgg<br>10801 caaaagcacc gccggacatc agcgctagcg gagtgtatac tggcttacta tgttggcact<br>10861 gatgagggtg tcagtgaagt gcttcatgtg gcaggagaaa aaaggctgca ccggtgcgtc<br>10921 agcagaatat gtgatacagg atatattccg cttcctcgct cactgactcg ctacgctcgg<br>10981 tcgttcgact gcggcgagcg gaaatggctt acgaacgggg cggagatttc ctggaagatg<br>11041 ccaggaagat acttaacagg gaagtgagag ggccgcggca aagccgtttt tccataggct<br>11101 ccgcccccct gacaagcatc acgaaatctg acgctcaaat cagtggtggc gaaacccgac<br>11161 aggactataa agataccagg cgtttccccc tggcggctcc ctcgtgcgct ctcctgttcc<br>11221 tgcctttcgg tttaccggtg tcattccgct gttatggccg cgtttgtctc attccacgcc<br>11281 tgacactcag ttccgggtag gcagttcgct ccaagctgga ctgt
pTarget	1 gtttgacagc ttatcatcga ctgcacggtg caccaatgct tctggcgtca ggcagccatc<br>61 ggaagctgtg gtatggctgt gcaggtcgta aatcactgca taattcgtgt cgctcaaggc<br>121 gcactcccgt tctggataat gtttttttgcg ccgacatcat aacggttctg gcaaatattc<br>181 tgaaatgagc tgttgacaat taatcatccg gctcgtataa tgtgtggaat tgtgagcgga<br>241 taacaatttc acacaggaaa cagcgccgct gagaaaaagc gaagcggcac tgctctttaa<br>301 caatttatca gacaatctgt gtgggcactc gaccggaatt atcgattaac tttattatta<br>361 aaaattaaag aggtatatat taatgtatcg attaaataag gaggaataaa ccatgggggg<br>421 ttctcatcat catcatcatc atggtatggc tagcatgact ggtggacagc aaatgggtcg<br>481 ggatctgtac gacgatgacg ataaggatcc aaccctttttc caagcttgca acttcattaa<br>541 gtggaacagg ttctgtaaat gcaagagcag catgcttcca aggcgaattc gaagcttggc<br>601 tgttttggcg gatgagagaa gattttcagc ctgatacaga ttaaatcaga acgcagaagc<br>661 ggtctgataa aacagaattt gcctggcggc agtagcgcgg tggtcccacc tgaccccatg<br>721 ccgaactcag aagtgaaacg ccgtagcgcc gatggtagtg tggggtctcc ccatgcgaga<br>781 gtagggaact gccaggcatc aaataaaacg aaaggctcag tcgaaagact gggcctttcg

	841 ttttatctgt tgtttgtcgg tgaacgctct cctgagtagg acaaatccgc cgggagcgga<br>901 tttgaacgtt gcgaagcaac ggcccggagg gtggcgggca ggacgcccgc cataaactgc<br>961 caggcatcaa attaagcaga aggccatcct gacggatggc ctttttgcgt ttctacaaac<br>1021 tctttttgttt atttttctaa atacattcaa atatgtatcc gctcatgaga caataaccct<br>1081 gataaatgct tcaataatat tgaaaaagga agagtatgag tattcaacat ttccgtgtcg<br>1141 cccttattcc cttttttgcg gcattttgcc ttcctgtttt tgctcaccca gaaacgctgg<br>1201 tgaaagtaaa agatgctgaa gatcagttgg gtgcacgagt gggttacatc gaactggatc<br>1261 tcaacagcgg taagatcctt gagagttttc gccccgaaga acgttttcca atgatgagca<br>1321 cttttaaagt tctgctatgt ggcgcggtat tatcccgtgt tgacgccggg caagagcaac<br>1381 tcggtcgccg catacactat tctcagaatg acttggttga gtactcacca gtcacagaaa<br>1441 agcatcttac ggatggcatg acagtaagag aattatgcag tgctgccata accatgagtg<br>1501 ataacactgc ggccaactta cttctgacaa cgatcggagg accgaaggag ctaaccgctt<br>1561 ttttgcacaa catgggggat catgtaactc gccttgatcg ttgggaaccg gagctgaatg<br>1621 aagccatacc aaacgacgag cgtgacacca cgatgcctgt agcaatggca acaacgttgc<br>1681 gcaaactatt aactggcgaa ctacttactc tagcttcccg gcaacaatta atagactgga<br>1741 tggaggcgga taaagttgca ggaccacttc tgcgctcggc ccttccggct ggctggttta<br>1801 ttgctgataa atctggagcc ggtgagcgtg ggtctcgcgg tatcattgca gcactggggc<br>1861 cagatggtaa gccctcccgt atcgtagtta tctacacgac ggggagtcag gcaactatgg<br>1921 atgaacgaaa tagacagatc gctgagatag gtgcctcact gattaagcat tggtaactgt<br>1981 cagaccaagt ttactcatat atactttaga ttgatttaaa acttcatttt taatttaaaa<br>2041 ggatctaggt gaagatcctt tttgataatc tcatgaccaa aatcccttaa cgtgagtttt<br>2101 cgttccactg agcgtcagac cccgtagaaa agatcaaagg atcttcttga gatccttttt<br>2161 ttctgcgcgt aatctgctgc ttgcaaacaa aaaaaccacc gctaccagcg gtggtttgtt<br>2221 tgccggatca agagctacca actctttttc cgaaggtaac tggcttcagc agagcgcaga<br>2281 taccaaatac tgtccttcta gtgtagccgt agttaggcca ccacttcaag aactctgtag<br>2341 caccgcctac atacctcgct ctgctaatcc tgttaccagt ggctgctgcc agtggcgata<br>2401 agtcgtgtct taccgggttg gactcaagac gatagttacc ggataaggcg cagcggtcgg<br>2461 gctgaacggg gggttcgtgc acacagccca gcttggagcg aacgacctac accgaactga<br>2521 gatacctaca gcgtgagcta tgagaaagcg ccacgcttcc cgaagggaga aaggcggaca<br>2581 ggtatccggt aagcggcagg gtcggaacag gagagcgcac gagggagctt ccagggggaa<br>2641 acgcctggta tctttatagt cctgtcgggt ttcgccacct ctgacttgag cgtcgatttt<br>2701 tgtgatgctc gtcagggggg cggagcctat ggaaaaacgc cagcaacgcg gcctttttac<br>2761 ggttcctggc cttttgctgg ccttttgctc acatgttctt tcctgcgtta tcccctgatt<br>2821 ctgtggataa ccgtattacc gcctttgagt gagctgatac cgctcgccgc agccgaacga<br>2881 ccgagcgcag cgagtcagtg agcgaggaag cggaagagcg cctgatgcgg tattttctcc<br>2941 ttacgcatct gtgcggtatt tcacaccgca tatggtgcac tctcagtaca atctgctctg<br>3001 atgccgcata gttaagccag tatacactcc gctatcgcta cgtgactggg tcatggctgc<br>3061 gccccgacac ccgccaacac ccgctgacgc gccctgacgg gcttgtctgc tcccggcatc<br>3121 cgcttacaga caagctgtga ccgtctccgg gagctgcatg tgtcagaggt tttcaccgtc<br>3181 atcaccgaaa cgcgcgaggc agcagatcaa ttcgcgcgcg aaggcgaagc ggcatgcatt<br>3241 tacgttgaca ccatcgaatg gtgcaaaacc tttcgcggta tggcatgata gcgcccggaa<br>3301 gagagtcaat tcagggtggt gaatgtgaaa ccagtaacgt tatacgatgt cgcagagtat<br>3361 gccggtgtct cttatcagac cgtttcccgc gtggtgaacc aggccagcca cgtttctgcg<br>3421 aaaacgcggg aaaaagtgga agcggcgatg gcggagctga attacattcc caaccgcgtg<br>3481 gcacaacaac tggcgggcaa acagtcgttg ctgattggcg ttgccacctc cagtctggcc<br>3541 ctgcacgcgc cgtcgcaaat tgtcgcggcg attaaatctc gcgccgatca actgggtgcc<br>3601 agcgtggtgg tgtcgatggt agaacgaagc ggcgtcgaag cctgtaaagc ggcggtgcac<br>3661 aatcttctcg cgcaacgcgt cagtgggctg atcattaact atccgctgga tgaccaggat<br>3721 gccattgctg tggaagctgc ctgcactaat gttccggcgt tatttcttga tgtctctgac<br>3781 cagacaccca tcaacagtat tattttctcc catgaagacg gtacgcgact gggcgtggag<br>3841 catctggtcg cattgggtca ccagcaaatc gcgctgttag cgggcccatt aagttctgtc<br>3901 tcggcgcgtc tgcgtctggc tggctggcat aaatatctca ctcgcaatca aattcagccg<br>3961 atagcggaac gggaaggcga ctggagtgcc atgtccggtt ttcaacaaac catgcaaatg<br>4021 ctgaatgagg gcatcgttcc cactgcgatg ctggttgcca acgatcagat ggcgctgggc<br>4081 gcaatgcgcg ccattaccga gtccgggctg cgcgttggtg cggatatctc ggtagtggga<br>4141 tacgacgata ccgaagacag ctcatgttat atcccgccgt taaccaccat caaacaggat<br>4201 tttcgcctgc tggggcaaac cagcgtggac cgcttgctgc aactctctca gggccaggcg<br>4261 gtgaagggca atcagctgtt gcccgtctca ctggtgaaaa gaaaaaccac cctggcgccc<br>4321 aatacgcaaa ccgcctctcc ccgcgcgttg gccgattcat taatgcagct ggcacgacag<br>4381 gtttcccgac tggaaagcgg gcagtgagcg caacgcaatt aatgtaagtt agcgcgaatt<br>4441 gatctg	
Name	Sequence (5'-3')	Description
Gene fragments		

dPalm fragment	GCCAAAACCATCGGCCTGTCGCGCCAGATACACCTGTTTTTCA<br>CTCTGTGGCTGCCGCATCTGTGCCGCAAAGACCCGCGTTTTGC<br>CAACACCTACACCGTGTTCGCCGCAGCGGCCGCATTCTTTTTG<br>ATTGGCCCGTGGCGCAGCCAGCAAACGCTGGCGCTAACAATGG<br>CGCAGGACTTCGCGCGCTACAGCGGCCACAATCCGGCGCTGCA<br>TTTTTCACTCGGGCTGGTGCAGGCCAAACCCGGCTACCCGGTG<br>CGGGCGCTGGCGGCTCAGGCCGAAGCCGCTCTGAAGCAGGCCA<br>AGCAACACCCCGGCAAGAACGCCATCTGCCTGTATAACGAGGT<br>GATGGGCTGGCCGGAATATGACGCGTTACTGGCGTGCAGTGAG<br>GAGCTGGCGCGCTGGCGCGAGCATGACGGCTACCCGCTCTCCT<br>CCGGCCTGCTTTACCGGTTGCTGGCGCTCAGTGAACAGTCGGC<br>GGAGGAGTCAGAAAAACCGCAGGCGGCACTGTGGCGCAGCCGG<br>CTGGCCTATTTTTTACGTCGCAATCTGGTGGATAACGTGAAAG<br>TCCCGACGAAAGAAAACCCGGCAGCGTTCCGCCATCAGCTTCA<br>TTTGGAATTGTTTGAGAAACTTGAGCAGCACCTGAAACGGCAC<br>CGCCAGCGTTACCGGGTGGCGCTTCAACGCCATCTCTACCACT<br>ACCGCACGGTGCCGCGCGGCGAGTAACATAT	Mutates GGDD motif of Cas10 Palm2 domain to AAAA
dCsm3 fragment	ATGCAACTGAACAATATCCAGACGTTACGCGCCACTCTGGTG<br>TGTGAAACCGGGTTACACATTGGCGGCGGCGACACCGCGTTG<br>CAGATTGGCGGCATCGCCAGCGCCGTGGTGCGCCACCCGTTG<br>ACCCAGCAACCTTACATTCCCGGCTCCAGCCTGAAAGGCAAA<br>CTGCGCAGCCTGCTCGAATGGCGCGCTGGTGTGGTCGGCGAT<br>ACCGAGGGCAAGGTGCTCAGTCATCAGGTTTACCAGCAACTG<br>ATCGACGATAAAAAACAGGCACAGGCACTACAGGCACTGAAA<br>ATCCTGCAACTGTTTGGCGTCAGCGGCGGCGACAAGCTTTCC<br>GCCGAACAAGCCCAACAGATCGGCCCGACTCGCCTGTCCTTC<br>TGGGATTGCGAATTTGACGAACACTGGCTGGCGCAGCAAGGC<br>GGGCGCGTCCAGACCGAAGAGAAAGCGGAAAACTGTATTGAC<br>CGCATCAGTGGCGTGGCGCTGCACCCGCGCTTTATCGAGCGT<br>GTGCCCGCCGGCAGCCGCTTTGACTTTCGCCTCACCGTGCGC<br>CAGCTTGATGGCGACAGCCCCGACCTGCTCGACACCCTGTTG<br>GCGGGCCTGAAAATGCTGGAGCTGGACGGGCTAGGCGGCAGC<br>ATTTCCCGTGGGTATGGCAAAGTGCGCTTTGAAGCGCTGACC<br>CTCGACGGGAAAGATCTCCAGCCGCGCTTTGAGCAGTTACAG<br>CCGTTTAAACACACCACGCAGGGAGCCGGATGA	Introduces D34A mutant to Csm3
B01 fragment	TAATACGACTCACTATAGGGAGACCATGGGTCCTTACGGACG<br>CTCCCTGACTGAAGGGATTAAGACTCCGGAGTCAGATGCACG<br>ATGGTGTCTGTTTGAGGTCCTTACGGACGCTCCCTGACTGAAGG<br>GATTAAGACCTCGAGGCTGTGGTCTAGACA	Cloning spacer B01, for expression of crRNA-B01
S01 fragment	5’TAATACGACTCACTATAGGGAGACCATGGGTCCTTACGGACGCT<br>CCCTGACTGAAGGGATTAAGACACAGGAGTCAGATGCACGATGGT<br>GTCTGTTTGAGGTCCTTACGGACGCTCCCTGACTGAAGGGATTAAG<br>ACCTCGAGGCTGTGGTCTAGACA	Cloning spacer S01, for expression of crRNA-S01
Oligonucleotides		

prCCP_077	AATTCTGTTTTATCAGACCGTTATTCCAGACTATCTATATACACCCGCC	Rev primer to add pBAD homology to NucC
prCCP_077	AATTCTGTTTTATCAGACCGTTATTCCAGACTATCTATATACACCCGCC	Rev primer to add pBAD homology to NucC
prCCP_078	GGCTAACAGGAGGAATTAACATGACTAATCAGGCAAAAAAGTTATCTAGAAT	Fwd primer to add pBAD homology to NucC
prCCP_079	GTTAATTCCTCCTGTTAGCCCAAAAAACGG	Rev primer to amplify region of interest from pBAD
prCCP_080	CGGTCTGATAAAACAGAATTTGCCTGGC	Fwd primer to amplify region of interest from pBAD
prCCP_085	GCAGCAGTGATTTTTGACCGGCATTACACA	Rev primer to introduce D83Q mutation in NucC
prCCP_086	TATCTGGTCGCTGGTCTGGC	Fwd primer to introduce D83Q mutation in NucC
prCCP_087	GCAGTTAAACCAACCATTAATAAAACCTACCTTGAATATG	Rev primer to introduce E114A mutation in NucC
prCCP_088	CAGTACCGCGTACACCGCCTC	Fwd primer to introduce E114A mutation in NucC
prCCP_101	CCACGCAGGGAGCCGGATGA	Rev primer to copy pACYC-Csmregion of interest for Gibson assembly
prCCP_102	TGGATATTGTTCAGTTGCATTATTTACCTCCTTTATGA	Fwd primer to copy pACYC-Csm region of interest for Gibson assembly
prCCP_103	GTGCCGCGCGGCGAGTAACATATG	Rev primer to copy pACYC-Csm region

		of interest for Gibson assembly
prCCP_104	CGACAGGCCGATGGTTTTGGC	Fwd primer to copy pACYC-Csm region of interest for Gibson assembly
prCCP_109	AGCGACGCAATAGATGCAGT	Fwd primer to introduce Q81A into pBAD NucC
prCCP_110	GGTCTGGCCTTCTGAATCCA	Rev primer to introduce Q81A into pBAD NucC
prCCP_121	CGTTCCGTTGAAGCGCCTAGGTC	Rev primer to make pACYC-Csm ΔNucC
prCCP_122	GAATAACTCGAGAGGTTACAGCCTGCATAAT	Fwd primer to make pACYC-Csm ΔNucC
prCCP_185	CACCGCGTTGGCGATTGGCGGCATCG	Fwd primer to make Q29A Csm3
prCCP_186	TCGCCGCCGCCAATGTGT	Rev primer to make Q29A Csm3
prCCP_187	CGACACCGCGGCGCAGATTGGCG	Fwd primer to make L28A Csm3
prCCP_188	CCGCCGCCAATGTGTAAC	Rev primer to make L28A Csm3
prCCP_189	GCGGGCGGCATCGACAGCG	Fwd primer to make I30A Csm3
prCCP_190	CTGCAACGCGGTGTCG	Rev primer to make I30A Csm3
prCCP_191	TGCGCTGTTAGCGAACCTGAAACCGC	Fwd primer to make H17A Cas10
prCCP_192	AAGGCAGCCACATGG	Rev primer to make H17A Cas10
prIVT_001	GAATTCTAATACGACTCACTATAGGGCAACTTCATTAAGTGGAA	Primer for overlap extension PCR to generate mismatch targets

prIVT_002	TACTTCCGCATTTACAGAACCTGTTCCACTTAATGAAGTTGC	Pairs with prIVT_001 to generate cognate target
prIVT_003	TACTTCCGGATTTACAGAACCTGTTCCACTTAATGAAGTTGC	Pairs with prIVT_001 to generate +1 mismatch
prIVT_004	TACTTCCGCTTTTACAGAACCTGTTCCACTTAATGAAGTTGC	Pairs with prIVT_001 to generate +2 mismatch
prIVT_005	TACTTCCGCAATTACAGAACCTGTTCCACTTAATGAAGTTGC	Pairs with prIVT_001 to generate +3 mismatch
prIVT_006	TACTTCCGCATATACAGAACCTGTTCCACTTAATGAAGTTGC	Pairs with prIVT_001 to generate +4 mismatch
prIVT_007	TACTTCCGCATTAACAGAACCTGTTCCACTTAATGAAGTTGC	Pairs with prIVT_001 to generate +5 mismatch
prIVT_008	TACTTCCGCATTTTCAGAACCTGTTCCACTTAATGAAGTTGC	Pairs with prIVT_001 to generate +6 mismatch
prIVT_009	TACTTCCGCATTTAGAGAACCTGTTCCACTTAATGAAGTTGC	Pairs with prIVT_001 to generate +7 mismatch
prIVT_010	TACTTCCGCATTTACTGAACCTGTTCCACTTAATGAAGTTGC	Pairs with prIVT_001 to generate +8 mismatch
prIVT_011	TACTTCCGCATTTACACAACCTGTTCCACTTAATGAAGTTGC	Pairs with prIVT_001 to generate +9 mismatch
prIVT_012	TACTTCCGCATTTACAGTACCTGTTCCACTTAATGAAGTTGC	Pairs with prIVT_001 to generate +10 mismatch
prIVT_013	TACTTCCGCATTTACAGATCCTGTTCCACTTAATGAAGTTGC	Pairs with prIVT_001 to generate +11 mismatch
ssRNA-d02	GCAACUUCAUUAAGUGGAACAGGUUCUGUAAAUGCGGAAGUA	

Cognate		
ssRNA-d03 Non-cognate	GCAACUUCAUUAAGUGGAACAGGUUCUGUAAAUGGUCUUAAU	
HBB target	CUCAAACAGACACCAUGGUGCAUCUGACUCCUGCGGAGAAGUCUGCCG	Synthetic RNA mimicking *HBB* transcript
A20T target	CUCAAACAGACACCAUGGUGCAUCUGACUCCUGAGGAGAAGUCUGCCG	Synthetic RNA mimicking the A20T transcript
Reporter DNA 1	/56-FAM/TCAAAGGCGCCCTTGGTACATACACGATTT	Cleaved by NucC in fluorogenic assay
Reporter DNA 2	AAATCGTGTATGTACCAAGGGCGCCTTTGA/31ABkFQ/	Cleaved by NucC in fluorogenic assay

Table S2. Buffers used in Cas10-Csm purification

Name	Reagent
**Phosphate lysis buffer**	100 mM NaH_2PO_4 pH 8.0
	600 mM NaCl
	20 mM imidazole
	1 mg/mL lysozyme
	2 mM PMSF
	0.1% (v/v) triton X-100
	1% v/v glycerol
	1 mM DTT
	500 µM EDTA
**Phosphate wash buffer 1**	100 mM NaH_2PO_4
	150 mM NaCl
	1 mM DTT
	1% (v/v) glycerol
	20 mM imidazole
**Phosphate wash buffer 2**	Phosphate wash buffer 1 with 40 mM imidazole
**Phosphate elution buffer 1**	Phosphate wash buffer 1 with 100 mM imidazole
**Phosphate elution buffer 2**	Phosphate wash buffer 1 with 250 mM imidazole
**Tris lysis buffer**	50 mM Tris-HCl pH 8.0
	300 mM NaCl
	20 mM imidazole
	1 mg/ml lysozyme
	1 mM PMSF
	0.1% (v/v) triton X-100
**Tris wash buffer**	Tris lysis buffer with 40 mM imidazole and 10% (v/v) glycerol
**Tris elution buffer 1**	Tris lysis buffer with 100 mM imidazole and 10% (v/v) glycerol
**Tris elution buffer 2**	Tris lysis buffer with 250 mM imidazole and 10% (v/v) glycerol
**Storage buffer**	50 mM Tris HCl pH 8.0
	20 mM NaCl
	5% (v/v) glycerol
**Reaction buffer 1**	50 mM Tris HCl pH 8.0
	150 mM NH_4Cl
	5% (v/v) glycerol
	10 mM $MgCl_2$
**Reaction buffer 2**	50 mM Tris HCl pH 8.0
	150 mM NaCl
	5% (v/v) glycerol
	10 mM $MgCl_2$
**Reaction buffer 3**	12.5 mM Tris HCl pH 8.0
	20 mM NaCl
	10 mM $MgCl_2$
	10% v/v glycerol
**EM buffer**	50 mM Tris HCl pH 8.0
	100 mM NaCl
	1 mM $MgSO_4$
	4 mM $CaCl_1$

Table S3. Mass spectrometry identification of proteins in SmCas10-Csm ultracentrifugation fractions.

Protein	Theoretical mass (kDa)	UNIPARC Identifiers	Unique peptide counts	PSMs[a]	Sum PEP score
**Cas10**	90.2	UPI0003926B6B	29	358	80.165
**Csm5**	63	UPI0003921A6A	21	431	80.144
**ArnA**	74.2	UPI0000000F69	26	96	64.091
**Csm3**	27.2	UPI00039276A8	19	892	53.839
**Csm2**	15.8	UPI000392639F	13	583	39.57
**Csm4**	35.7	UPI001F2BC94D	10	219	39.294
**GroEL**	57.3	UPI0000000ED4	14	32	30.367
**AdhE**	96.1	UPI0000000054	16	37	29.725
**SlyD**	20.8	UPI0000135A43	5	19	25.797

[a] All proteins comprising 1% or more of total protein as determined by number PSM are reported.
PSM - Peptide spectrum match
PEP - Posterior error probability for peptide spectrum matches, PEP score is the negative logarithm of the PEP value

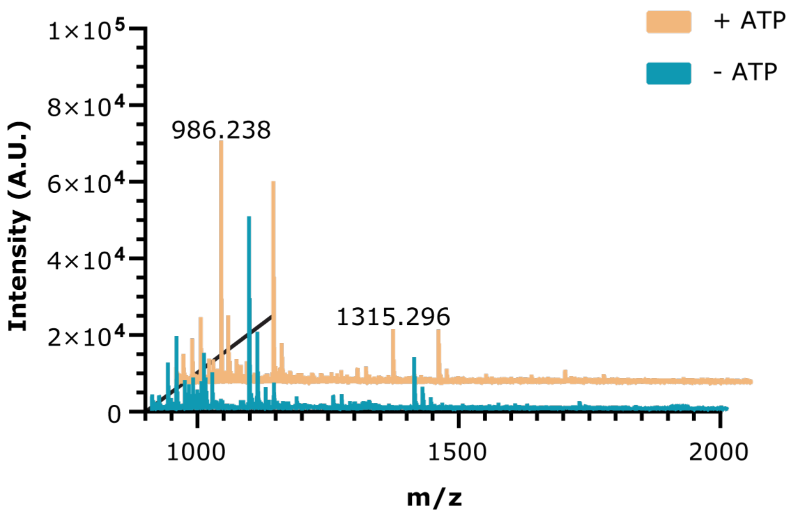

Figure S3. MALDI mass spectrometry of cOA synthesis reactions plus and minus ATP.

Table S4. Cryo-EM Statistics for Data Collection and Model Quality

**Data collection and processing**	**Cas10-Csm unbound to target RNA**<br>**9ZS2**<br>**EMD-74675**	**Cas10-Csm bound to target RNA**<br><br>**9ZS4**<br>**EMD-74688**
Magnification	150,000	150,000
Voltage (kV)	200	200
Electron exposure ($e^-/Å^2$)	40.0	40.0
Defocus range (μm)	0.1-3.5	0.1-3.0
Pixel size (Å)	0.94	0.94
Initial particles (no.)	593,739	324,865
Final particles (no.)	42,471	129,762
Map resolution (Å)	4.4	3.8
FSC threshold	0.143	0.143
Map sharpening B factor ($Å^2$)	118.6	110.9
**Refinement**		
Model resolution (Å)	4.4	3.8
FSC threshold	0.143	0.143
Model composition		
Nonhydrogen atoms	18,543	23,918
Protein residues	2308	2867
RNA residues	30	64
Bonds (RMSD)		
Bond lengths (Å)	0.003	0.003
Bond angles (°)	0.770	0.742
Validation		
Molprobity score	1.99	2.00
Clashscore	15.68	9.85
Ramachandran plot (%)		
Outliers	0.22	0.00
Allowed	4.02	3.34
Favored	95.76	96.66
B factors, mean ($Å^2$)		
Protein	420.62	203.32
RNA	313.83	148.32

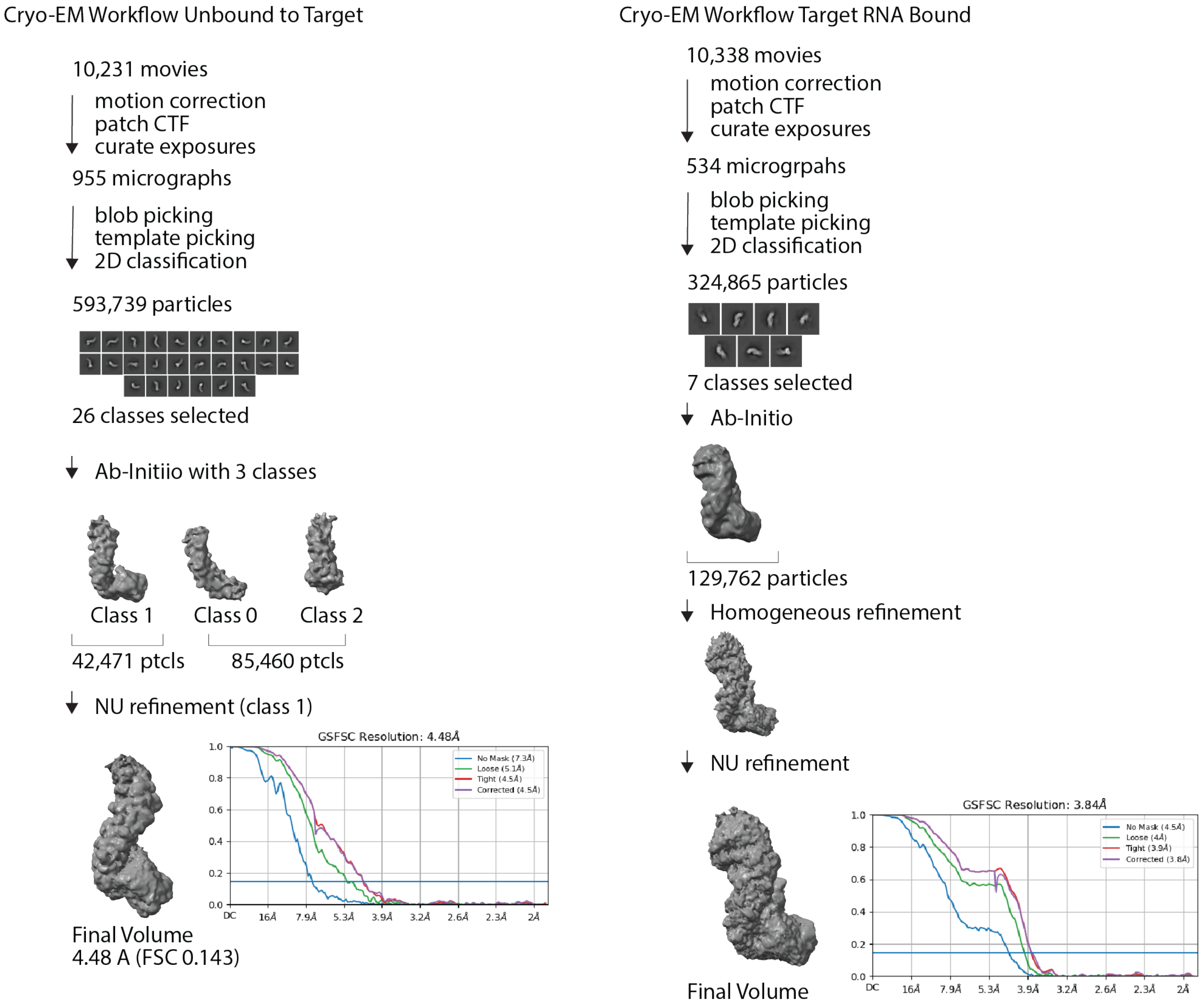

Figure S4. Workflow for single particle reconstructions of SmCas10-Csm by cryo-EM.

Table S5. Fluorescence signal for titrations of wild type *HBB* RNA and variant

crRNA-B01/wt *HBB* RNA

Concentration	Raw fluorescence		Background subtracted intensity	I/σ(I)
	**Mean**	**σ**	**Mean**	
**1 uM**	11459	541	8926	16.5
**100 nM**	10698	783	8166	10.4
**10 nM**	11084	1163	8551	7.4
**1 nM**	4036	267	1504	5.6
**100 pM**	2624	203	92	0.5
**10 pm**	2520	147	-12	-0.1
**1 pm**	2382	215	-151	-0.7
	Blank mean 2533	Blank s = 197		

crRNA-B01/A20T RNA

Concentration	Raw fluorescence		Background subtracted intensity	I/σ(I)
	**Mean**	**σ**	**Mean**	
**1 uM**	10496	769	7986	10.4
**100 nM**	4219	492	1709	3.5
**10 nM**	2708	152	198	1.3
**1 nM**	2397	168	-113	-0.7
**100 pM**	2624	120	115	1.0
**10 pm**	2066	105	-444	-4.2
**1 pm**	2608	122	98	0.8
	Blank mean 2509	Blank s = 266		

crRNA-S01/A20T RNA

Concentration	Raw Fluorescence		Background subtracted intensity	I/σ(I)
	**Mean**	**σ**	**Mean**	
**1 uM**	15129	162	14013	86.6
**100 nM**	15069	345	13952	40.5
**10 nM**	13574	2133	12458	5.8
**1 nM**	9623	1836	8507	4.6
**100 pM**	1794	390	678	1.7
**10 pm**	1217	151	101	0.7
**1 pm**	1198	187	82	0.4
	Blank mean 1116	Blank s = 191		

crRNA-S01/wt *HBB* RNA

Concentration	Raw Fluorescence		Background subtracted intensity	I/σ(I)
**1**	**Mean**	**σ**	**Mean**	
**1 uM**	5959	492	4760	9.7
**100 nM**	6402	711	5204	7.3
**10 nM**	1725	233	527	2.3
**1 nM**	1197	169	-1	0.0
**100 pM**	1074	253	-124	-0.5
**10 pm**	1216	134	18	0.1
**1 pm**	1406	460	208	0.5
	Blank mean 1198	Blank s = 76		

I/σ(I), mean of background subtracted intensity divided by standard deviation of this mean.

Table S6. Fluorescence signal for SNP detection in contrived samples.

crRNA-B01

**Targets**	**Raw fluorescence**		**Background subtracted intensity**	**I/ σ(I)**
	**Mean**	**σ**	**Mean**	
**HBB**	13314	293	11585	39.5
**HBB / A20T**	11645	131	9916	75.6
**A20T**	4292	566	2564	4.5
	Blank mean 1729	Blank s = 143		

crRNA-S01

**Targets**	**Raw fluorescence**		**Background subtracted intensity**	**I/ σ(I)**
	**Mean**	**σ**	**Mean**	
**HBB**	3702	451	174	0.4
**HBB / A20T**	12817	360	9288	25.8
**A20T**	12010	88	8481	96.7
	Blank mean 3529	Blank s = 414		
